# Melanoma-associated antigen A4: A cancer/testis antigen as a target for adoptive T-cell receptor T-cell therapy

**DOI:** 10.1016/j.ctrv.2025.102891

**Published:** 2025-01-26

**Authors:** Gabrielle Knafler, Alan L. Ho, Kathleen N. Moore, Seth M. Pollack, Jean-Marc Navenot, Joseph P. Sanderson

**Affiliations:** a Envision Pharma Group Fairfield CT USA; b Department of Medicine, Memorial Sloan Kettering Cancer Center, and Weill Medical College of Cornell University New York NY USA; c Stephenson Cancer Center, University of Oklahoma Health Sciences Center Oklahoma City OK USA; d Lurie Cancer Center, Department of Medicine, Northwestern University Feinberg School of Medicine Chicago IL USA; e Adaptimmune Philadelphia PA USA; f Adaptimmune Abingdon UK

**Keywords:** T-cell therapy, Melanoma-associated antigen, Cancer/testis antigen, Adoptive cell therapy, T-cell receptor therapy

## Abstract

T-cell receptor (TCR) T-cell therapies are adoptive cell therapies in which patient cells are engineered to express TCRs targeting specific cancer antigens and infused back into the patient. Since TCR recognition depends on antigen presentation by the human leukocyte antigen system, TCRs can respond to intracellular antigens. Cancer/testis antigens (CTAs) are a large family of proteins, many of which are only expressed in cancerous tissue and immune-privileged germline sites. Melanoma-associated antigen A4 (MAGE-A4) is an intracellular CTA expressed in healthy testis and placenta, and in a range of cancers, including esophageal, head and neck, gastric, ovarian, colorectal, lung, endometrial, cervical, bladder, breast and prostate cancers; soft tissue sarcomas; urothelial and hepatocellular carcinomas; osteosarcoma; and melanoma. This expression pattern, along with the immunogenicity and potential role in tumorigenesis of MAGE-A4 make it a prime target for TCR T-cell therapy. We outline the preclinical and clinical development of TCR T-cell therapies targeting CTAs for treatment of solid tumors, highlighting the need for extensive preclinical characterization of putative off-target, and potential on-target but off-tumor, effects. We identified ten clinical trials assessing TCR T-cell therapies targeting MAGE-A4. Overall, manageable safety profiles and signals of efficacy have been observed, especially in patients with advanced synovial sarcoma, myxoid/round cell liposarcoma, ovarian, head and neck, and urothelial cancers, with one TCR T-cell therapy approved by the US Food and Drug Administration in August 2024. We also review the limitations, and strategies to enhance efficacy and improve safety, of these therapies, and summarize related immunotherapies targeting MAGE-A4.

## Introduction

Immunotherapy has improved treatment options and outcomes for many people with cancer; however, responses are often limited in non-hematologic advanced solid tumors. Chimeric antigen receptor (CAR) T-cell therapies approved by the US Food and Drug Administration (FDA) for various hematologic malignancies target CD19 or BCMA cell surface antigens not expressed in solid tumors, and CAR T-cell therapies targeting a variety of antigens have shown limited efficacy for solid tumors. Weak affinity of endogenous T-cell receptors (TCRs) to their cognate tumor antigens dampens immune response, contributing to suboptimal anti-tumor activity. Adoptive CD4 + and CD8 + T-cell therapy with affinity-enhanced TCRs targeting tumor antigens expressed in any cellular compartment, including intracellularly, is rapidly developing in the immuno-oncology field, with promising preliminary clinical results reported. Several factors make TCR-based therapies a valuable addition to the adoptive cell therapy field. Owing to differences in signaling and effector mechanisms, TCRs are generally more sensitive than CARs [1], and may be able to penetrate solid tumors and engage the wider immune system more effectively. Their ability to target antigens from any cellular compartment means they can theoretically target a wider range of cancers. However, targeted tumor antigen selection is of vital importance to engineer safe/effective TCR T cells.

Tumor antigens can be categorized by expression patterns and resulting tumor specificity. Tumor-specific antigens are not present in normal tissue, arising during tumor development from mutations that generate novel peptide sequences [2] or from oncovirus-derived proteins [3]. Tumor-associated antigens (TAAs) are expressed in normal and diseased tissues at varying levels. Among TAAs, tissue differentiation antigens and overexpressed antigens have relatively low tumor specificity, whereas cancer/testis antigens (CTAs) have high tumor specificity. CTAs are generally restricted to germ cells and trophoblastic cells in normal tissue [4,5], but are overexpressed in many different tumor types.

CTAs were discovered in 1991 when cytotoxic T lymphocytes (CTLs) from a patient with melanoma recognized antigenic peptides on a human melanoma cell line encoded by a previously unknown gene, melanoma-associated antigen A1 (*MAGE-A1*) [6]. Since then, many CTAs, including other MAGE family proteins, have been described. We focus this review on the role of CTAs, and specifically of a MAGE family member, MAGE-A4, as promising targets for safe and effective cancer TCR T-cell therapy in the context of tumor expression and specificity, oncogenic activity and immunogenicity.

## Expression of CTAs

Many CTA genes show upregulated expression in cancer and germline-restricted expression in normal tissue. Over half of CTA genes are located on the X chromosome (referred to as CT-X), often members of multigene families [7], whereas others are distributed across the autosomes and Y chromosome in single copies (referred to as non-X CT) [8,9]. CT-X genes show restricted expression patterns, whereas non-X CT genes are more broadly expressed [8]. CTA gene and protein expression are generally correlated [10–12], with CTA messenger RNA (mRNA) levels > 1 % of testicular mRNA levels usually exhibiting protein expression [10].

CTAs are often co-expressed [13], and expression is characteristically heterogeneous among cancer types [14]. Cancers enriched for CTAs include melanoma and non-small cell lung carcinoma, followed by sarcomas, head and neck, ovarian, breast, colon and endometrial cancers, whereas renal cell carcinoma, glioblastoma, and leukemia tend to show low CTA enrichment [8,14].

Histological and molecular tumor subtypes show heterogeneous CTA expression [14]. Furthermore, even cancer types with overall low CTA expression can have specific subtypes enriched for CTAs. For example, PRAME is highly expressed in clear cell type B renal cell carcinoma [14]. Varying CTA expression by histological grade and/or clinical stage also occurs [11,13].

CTA expression within individual tumor samples can be uniform or non-uniform, with some tumors showing homogeneous CTA positivity and others showing positivity in only a small subset of cells [15]. For example, 15/19 and 11/13 lung cancer specimens showed < 50 % of cells positive for MAGE-1 or New York esophageal squamous cell carcinoma 1 (NY-ESO-1), respectively [16,17], whereas two of three synovial sarcoma (SyS) tumor [16] and 18/25 myxoid/round cell liposarcoma (MRCLS) samples showed homogeneous NY-ESO-1 protein expression [18].

Mechanisms regulating CTA-coding genes include epigenetic modifications. Cytosine DNA hypomethylation is associated with CTA gene expression [19]. DNA hypomethylation affects promoter regions of CTA genes [20], resulting in increased transcription, whereas hypermethylation represses transcription. *NY-ESO-1* and *PRAME* expression was increased in four chondrosarcoma cell lines, but not in healthy peripheral blood mononuclear cells, following treatment with a DNA methyltransferase inhibitor [21]. Additionally, inhibition of histone lysine deacetylation and mediators of histone lysine methylation that repress gene expression resulted in enhanced gene expression of CTAs in human cancer cells when combined with DNA-demethylating agents [22,23].

## Oncogenic role and immunogenicity of CTAs

The function of CTAs in relation to cancer is complex and likely depends on tumor type [24]. Some CTA expression in cancer may simply be a byproduct of epigenetic dysregulation and not result in any significant functional change. Alternatively, CTAs may be oncogenic, tumor suppressive or both, either inducing or inhibiting cancer cell proliferation, apoptosis and metastases.

Many CTAs are immunogenic, especially CT-X [25]. As evidenced by their discovery involving CTLs and later development of the SEREX methodology to screen for tumor antigens reacting with anti-tumor immunoglobulin G antibodies in patient serum [26,27], CTAs can induce cellular and humoral immune responses. Because CTAs are usually intracellular [15], they are presented to the immune system by the human leukocyte antigen (HLA) complex. An early study demonstrated concurrent antigen-specific humoral and cellular immune responses in a patient with melanoma showing high antibody production against NY-ESO-1- and HLA-restricted CTL reactivity against a tumor cell line positive for NY-ESO-1 [28]. More recently, cell-mediated and humoral immune responses against numerous CTAs in patients with hepatocellular carcinoma have been reported, signifying potential for further development of immunotherapeutic approaches to treat this solid cancer [29].

The immunogenicity and tumor-associated expression of CTAs make them attractive immunotherapy targets. DNA, RNA and peptide vaccines targeting several CTAs, most commonly NY-ESO-1 and the MAGE family, have been assessed in various clinical trials in a range of solid tumors, but have generally shown limited anti-tumor efficacy to date. This could be due to weak immunogenicity of chosen target antigens, inefficient antigen presentation by the tumor and/or immunosuppressive tumor microenvironment. Use of adjuvants and alternate delivery systems, such as nanomaterials, optimization of target antigens and combinations with immunomodulatory therapies, may all have potential to improve CTA-targeting anti-tumor vaccine efficacy [30]. Here, we focus on TCR T-cell therapy and related immunotherapeutics specifically targeting MAGE-A4.

## TCR T-cell therapy targeting CTAs

**Fig. 1.**
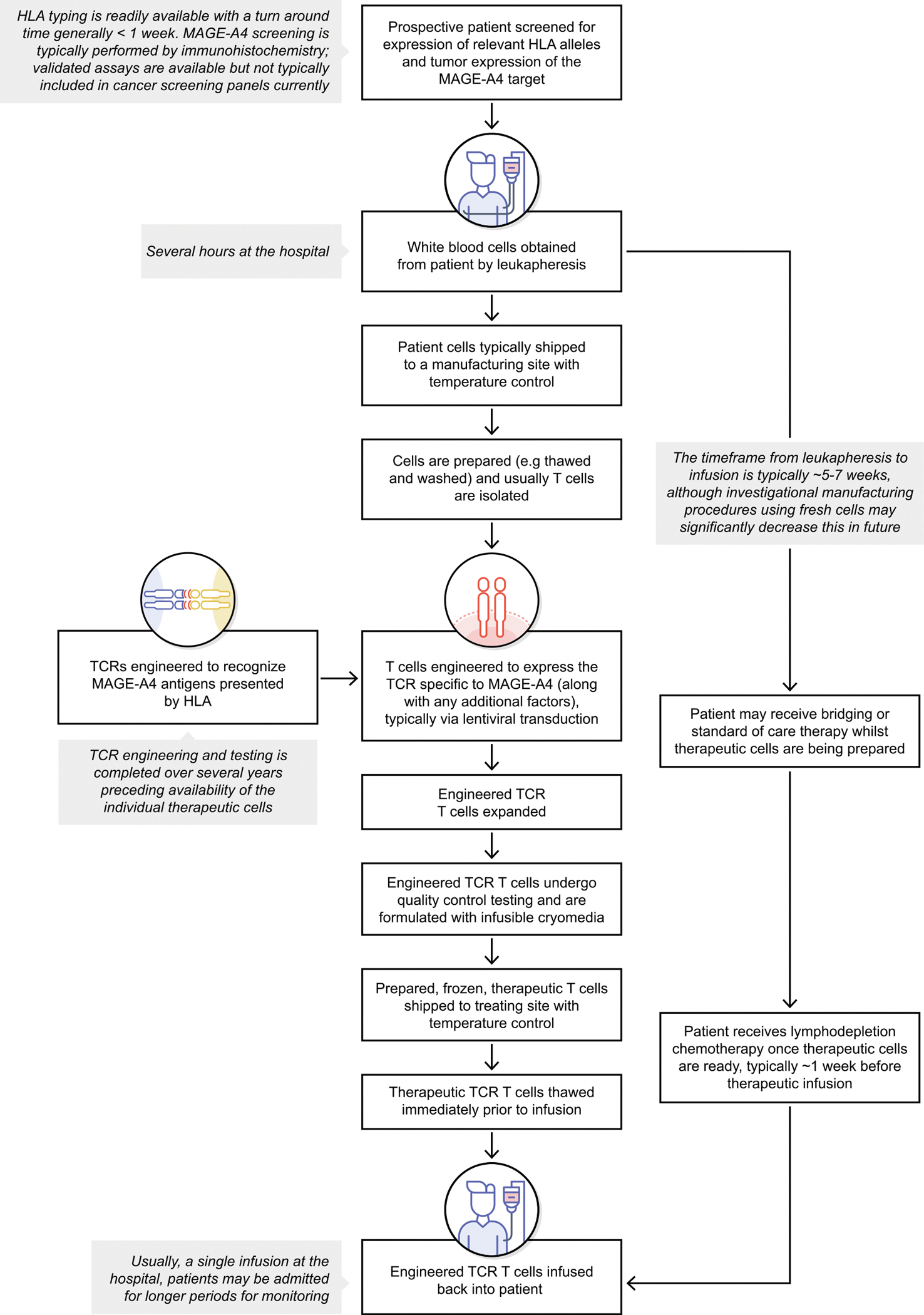
Representative treatment journey for engineered TCR T-cell therapies, with approximate timeframes indicated by italics; details may differ with different therapies. HLA: human leukocyte antigen; MAGE-A4: melanoma-associated antigen A4; TCR: T-cell receptor.

TCR T-cell therapy is an immunotherapy in which autologous T cells are collected from patients, genetically modified to express TCRs that target cancer-related antigens (including intracellular antigens), expanded *ex vivo* and infused back into patients (Fig. 1). Oncogenic CTAs are prime targets for TCR T-cell therapy, given their restricted expression in normal tissue, expression across several solid tumor types, potential role in cancer development and progression, and immunogenicity. Although immunogenicity may suggest germ and trophoblastic cell autoimmunity, classical HLA-A and HLA-B molecules are normally absent in germ and trophoblastic cells [31], precluding CTA presence on the surface of these cells, subsequent recognition by T cells and triggering of the immune response.

Numerous clinical trials employing TCR T-cell therapy targeting CTAs for treatment of different cancers are registered on ClinicalTrials.gov. NY-ESO-1 is the CTA targeted most, and clinical trials are more often in melanoma than in other cancers [32]. In addition, there are more phase I than phase I/II and II clinical trials, and most are ongoing rather than completed, indicating recent interest in this type of cancer immunotherapy.

Evidence of anti-tumor activity in solid tumors has been noted for TCR T-cell therapies. In a phase I/II trial with NY-ESO-1–directed T cells, best overall responses per Response Evaluation Criteria in Solid Tumors (RECIST) v1.1 of one complete response (CR), 14 partial responses (PRs), 24 stable disease (SD) and three progressive disease were observed in patients with SyS [33]. Two phase II pilot trials of TCR T-cell therapies targeting NY-ESO-1 reported clinical responses per RECIST in melanoma (four CRs, seven PRs) [34] and per independently assessed RECIST v1.1 in MRCLS (six PRs, 13 SD across two cohorts) [35]. A phase I trial of PRAME-directed T cells showed some level of disease control in 12 patients, whereas six patients (three with SyS, two with malignant melanoma, one with head and neck cancer) showed PRs per RECIST v1.1 [36]. A *meta*-analysis of efficacy of several different TCR-based adoptive cell therapy modalities (including tumor-infiltrating lymphocytes [TILs], CAR T cells and TCR T-cell therapies) in cutaneous melanoma showed an association with longer progression-free survival and likelihood of tumor response in patients treated with TCR T-cell therapies, especially those targeting NY-ESO-1 [37].

Safety findings have generally indicated manageable benefit-to-risk profiles. Most common adverse events (AEs) are cytokine release syndrome (CRS) resulting in systemic inflammation, and cytopenias due to pre–T-cell infusion lymphodepletion (LD) chemotherapy necessary to reduce competition among wild-type and engineered T cells [38]. Immune effector cell-associated neurotoxicity (ICANS), a common AE associated with CAR-T therapy [39], does not appear to be so common in TCR T-cell therapy of solid tumors. Despite the generally manageable side effect profile of TCR T-cell therapies, there have been more severe incidents. A phase I/II trial evaluating anti–MAGE-A3 TCR T-cell therapy in a range of metastatic cancers reported three patients who experienced neurotoxicity, resulting in two deaths. The TCR used in the study was modified by site-directed mutagenesis in the CDR3 region to increase its avidity and recognized MAGE-A3/A9/A12 epitopes, and further investigation revealed expression of MAGE-A12 in human brain tissue [40]. In other studies, two patients with late-stage and high-risk or relapsed melanoma treated with TCR T cells with mutations in the CDR2 region targeting MAGE-A3^a3a^ died after severe cardiac toxicity. The deaths were due to epitope recognition of a protein unrelated to CTAs that was expressed by contracting normal cardiac tissue [41,42]. Confirming absence of cross-reactive recognition of epitopes expressed in non-cancerous tissue is, therefore, essential to reduce off-target effects that may result in serious AEs, including fatalities.

## Rationale for developing MAGE-A4–targeted TCR T-cell therapies

The MAGE family is a group of conserved CTAs consisting of about 55 human genes, some of which are probable pseudogenes [43]. *MAGE-A,* −*B* and −*C* genes show germline and tumoral expression patterns characteristic of CTAs, whereas *MAGE-D,* −*E,* −*F,* −*G,* −*H* and −*L* are expressed in normal adult tissue [43]. MAGEs contain a highly conserved MAGE homology domain (MHD), 165–171 amino acids long, encoding two tandem winged-helix (WH-A and −B) motifs [44,45]. Although MAGE proteins are similar in overall MHD structure, they differ in relative orientation of WH-A and WH-B, potentially resulting in different unique cellular interactions [44,45].

**Fig. 2.**
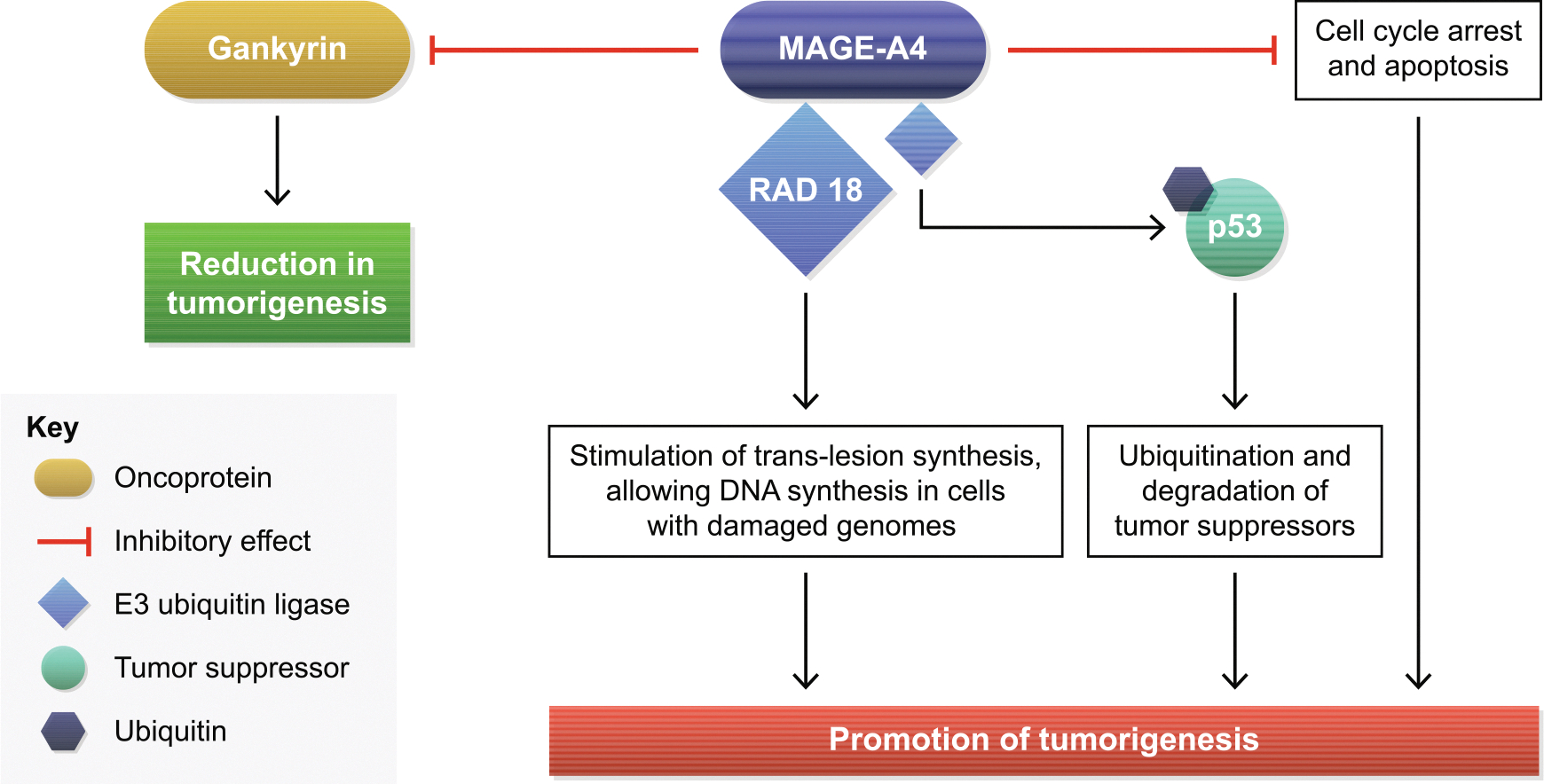
Potential mechanisms of MAGE-A4 in oncogenesis. MAGE-A4: melanoma-associated antigen A4.

The cellular role of MAGE-A4 is not fully understood. A yeast two-hybrid screen identified MAGE-A4 as a binding partner of the oncoprotein gankyrin, and MAGE-A4 suppressed gankyrin’s tumorigenic activity *in vitro* and in mouse models [46]. Other studies have shown MAGE proteins bind E3 ring ubiquitin ligases forming MAGE-RING ligases, which induce ubiquitination and subsequent proteasomal degradation of proteins, including tumor suppressors [45]. Multiple intracellular pathways are affected, possibly favoring tumorigenesis (Fig. 2). MAGE-A4 stabilizes the E3 ligase RAD18, resulting in stimulation of *trans*-lesion synthesis (TLS) [47]. TLS is a DNA damage-tolerance mechanism allowing DNA synthesis in cells with damaged genomes, contributing to tumorigenesis [48]. Furthermore, MAGE-A4 can promote growth of normal cells by preventing cell cycle arrest and inhibiting apoptosis [49], potentially contributing to tumor development by promoting survival. Support for this hypothesis was demonstrated when suppressing *MAGE-A* genes inhibited viability and induced apoptosis of a melanoma cell line [50]. These mechanisms describing the putative role of MAGE-A4 in oncogenesis and its role in chemoresistance suggest targeting MAGE-A4 could reduce tumor burden and sensitize cancer cells to chemotherapeutics [51].

**Fig. 3.**
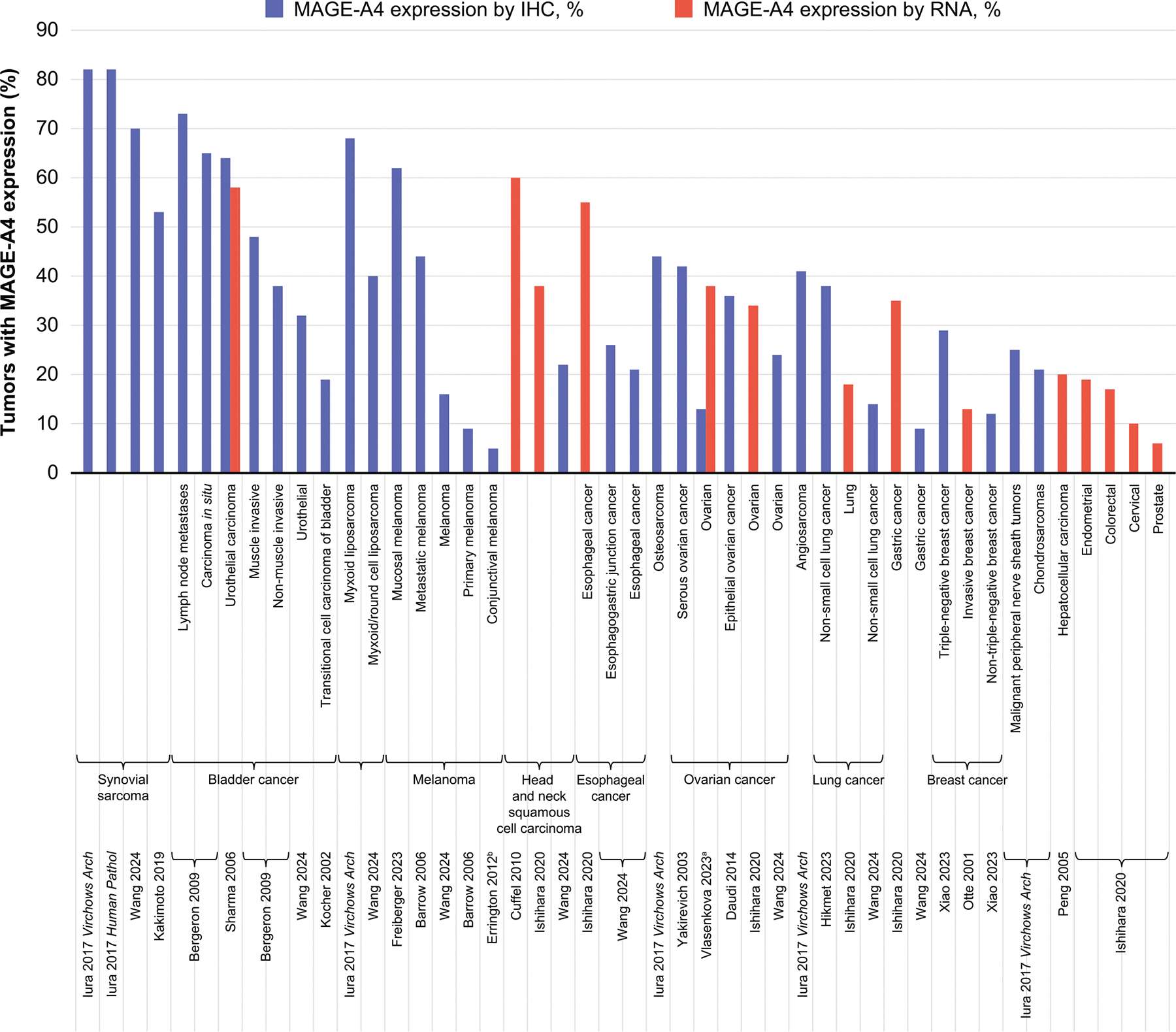
MAGE-A4 expression rates in various solid tumors. Expression rates are not directly comparable between studies due to different assay conditions and expression thresholds defining “positivity”. ^a^Reported as 25–50 % positive by RNA and 1–25 % positive by IHC. ^b^Reported as < 10 % by IHC. IHC: immunohistochemistry; MAGE-A4: melanoma-associated antigen A4.

MAGE-A4 expression has not been found in any healthy tissue apart from testis and placenta [5,52]. It is unlikely to be presented to the immune system at these sites due to varying mechanisms ensuring immune privilege, including absence of classical HLA-A expression [31]. However, MAGE-A4 is expressed in a wide range of cancers, including esophageal, head and neck, gastric, ovarian, colorectal, lung, endometrial, cervical, bladder, breast and prostate cancers; soft tissue sarcomas; urothelial and hepatocellular carcinomas; osteosarcoma; and melanoma [12,53–64]. To prospectively evaluate HLA and MAGE-A4 expression levels to identify potential patients suitable for MAGE-A4–targeted TCR T-cell therapy, an international screening study (NCT02636855) is ongoing in adult patients with advanced solid or hematologic malignancies. Data from this study were combined with data from the screening phase of the phase II SPEARHEAD-1 trial (NCT04044768), and of 1750 HLA-eligible patients with tumor samples assessed for MAGE-A4 expression by immunohistochemistry (IHC), 447 (26 %) expressed MAGE-A4 (defined with a cutoff of ≥ 30 % tumor cell staining at ≥ 2 + intensity) [52]. Fig. 3 shows an overview of the variety of expression levels and patterns from a range of studies.

There is no consensus regarding potential contribution of MAGE-A4 to prognosis. In some cancers, MAGE-A4 is associated with aggressive, metastatic tumors with poor prognosis. In a small series of soft tissue sarcomas, MAGE-A4 expression was positively correlated with tumor activity as measured by maximum standardized uptake value [65]. MAGE-A4 expression was also associated with high-grade and invasive phenotype bladder tumors [66], with poor survival in ovarian cancer [67] and poor outcomes independent of clinical parameters in head and neck squamous cell carcinoma [68]. MAGE-A expression by IHC and MAGE-A4 expression by RT-PCR was associated with malignant transformation of oral leukoplakia to squamous cell carcinoma [69], and MAGE-A4 expression was acquired with advancing disease in a large panel of primary and metastatic melanomas [70]. MAGE-A4 expression was correlated with higher histological grade in breast cancer, but expression of either MAGE-A4 or PRAME extended disease-free survival [60]. In some cancers, there are indications of tumor suppressor effects of MAGE-A4. In invasive ductal breast cancer and salivary gland carcinomas, MAGE-A4 levels were associated with better prognosis [71,72]. Interestingly, naturally acquired CD4 + T-cell responses against MAGE-A4 were detected in non-vaccinated patients with head and neck squamous cell carcinoma [73]. In non-small cell lung cancer, high expression of MAGE-A4 was associated with infiltration of CD163-positive macrophages and the FOXP3 marker, whereas low MAGE-A4 expression was associated with the CD3 pan–T-cell marker [12]. Taken together, these findings support developing MAGE-A4–targeted TCR T-cell therapies.

## Preclinical assessments of MAGE-A4–targeted TCR T-cell therapies

The potential for MAGE-A4–targeted immunotherapy as a promising strategy for treatment of solid malignancies led to several developments in TCR T-cell therapy. One study cloned TCR genes from an HLA-A*2402–restricted T-cell specific for MAGE-A4_143–151_ peptide, and retrovirally transduced them to polyclonally activated CD8 + T cells. These engineered TCR T cells showed *in vitro* cytotoxicity and interferon gamma (IFNγ) production, and long-term phenotypic, functional and molecular stability for > 6 months [74].

A subsequently developed TCR T-cell therapy, afamitresgene autoleucel (afami-cel; formerly ADP-A2M4), is composed of autologous CD4 + and CD8 + T cells that are transduced with an affinity-enhanced TCR recognizing the MAGE-A4_230–239_ peptide (GVYDGREHTV). Preclinical studies of afami-cel demonstrated IFNγ release, proliferation and cytotoxicity against MAGE-A4–positive tumor cell lines and primary tumor material *in vitro*. Afami-cel also showed dose-dependent anti-tumor effects in xenograft tumor mouse models. Extensive safety evaluation involving molecular analysis to predict other potential peptides recognized by afami-cel and 2D and 3D safety testing using human primary cells revealed no functionally relevant off-target and cross-reactivity [75].

An isolated TCR reactive to MAGE-A4 derived from primed T cells of an HLA-A*02–negative donor resulted in development of bbT485, an autologous TCR T-cell therapy. bbT485 demonstrated acceptable safety and enhanced anti-tumor activity *in vivo* compared with TCR T cells expressing a TCR derived from an HLA-A*02–positive donor. This increased potency may be due to the ability of bbT485 CD4 + T-helper cells to directly kill MAGE-A4–positive tumor cells [76].

Additional functional modifications to engineered T cells have also led to development of next-generation products. Uzatresgene autoleucel (uza-cel; formerly ADP-A2M4CD8) is an HLA-A*02–restricted mixed CD4 + and CD8 + T-cell therapy using the same MAGE-A4–targeting TCR as afami-cel, modified with an additional CD8α co-receptor designed to enhance functionality of CD4 + T cells. Improvements in T-cell activation, proliferation and cytokine production were noted, along with enhanced killing of antigen-expressing 3D tumor microspheres by engineered CD4 + T cells expressing CD8α co-receptor versus those without the co-receptor [77].

An HLA-A*24:02–restricted TCR T-cell therapy targeting MAGE-A4, including downregulation of endogenous TCR using small interfering RNA, has also been developed. These engineered T cells suppressed *in vivo* tumor growth. In a case study, a patient with uterine leiomyosarcoma who had CR following previous therapy had continued CR for > 3 years following two infusions, with results indicating long-term persistence of engineered T cells [78].

To support proliferation, survival and recruitment of engineered T cells, a MAGE-A4–targeted, HLA-A*02–restricted TCR T-cell therapy, ADP-A2M4N7X19, secretes interleukin 7 (IL-7) and C–C motif chemokine ligand 19 (CCL19). *In vitro* studies showed production of IL-7 by ADP-A2M4N7X19 improved functional response to repeated MAGE-A4 stimulation and increased MAGE-A4–dependent expansion. Additionally, CCL19 production by ADP-A2M4N7X19 led to immune cell migration [79]. Taken together, these preclinical studies demonstrating feasibility and potential of first- and next-generation MAGE-A4–targeted TCR T-cell therapies have led to development of larger-scale multicenter clinical trials in numerous indications.

## Clinical assessment of MAGE-A4–targeted TCR T-cell therapies

Table 1 summarizes completed and ongoing clinical trials assessing TCR T-cell therapies targeting MAGE-A4. One of the first clinical trials evaluating TCR T-cell therapies targeting MAGE-A4 in recurrent or metastatic esophageal carcinoma (UMIN000002395) showed minimal AEs in all ten HLA-A*24:02 patients, potentially due to lack of LD chemotherapy preceding TCR T-cell infusion. The phase I dose-escalating trial reported no patients experiencing any AEs for the first 14 days after infusion, with grade 1 skin reactions subsequently developing in four patients. Despite persistence of engineered T cells 1 to > 5 months after infusion and maintenance of antigen-specific tumor reactivity, tumor progression occurred in seven patients within 2 months after treatment. Three patients with minimal baseline tumor burden survived for > 27 months [80].

A phase I dose-escalation trial of safety and anti-tumor activity of afami-cel in 38 HLA-A*02-eligible patients with advanced MAGE-A4–expressing cancers across nine tumor types (NCT03132922) reported hematologic toxicities and generally low-grade CRS among other treatment-emergent AEs. There were two treatment-related fatalities: a 77-year-old patient with SyS, heavily pre-treated with chemotherapy, who died of aplastic anemia on day 55 of study, and a 71-year-old patient with ovarian cancer who died of an ischemic cerebrovascular accident on day 17 after grade 3 neurotoxicity. These patients received the highest-dose LD regimen of cyclophosphamide 1800 mg/m^2^ for 2 consecutive days with fludarabine 30 mg/m^2^ for 4 consecutive days. Following these deaths, high-dose cyclophosphamide LD regimen was discontinued in this and subsequent trials. Overall response rate (ORR) confirmed by RECIST version 1.1 was 24 % (9/38), with 7/16 (44 %) PRs in SyS and 2/22 PRs in all other cancers. Median duration of response (DOR) was 25.6 weeks overall (95 % confidence interval [CI] 12.286–not reached) and 28.1 weeks (95 % CI 12.286–not reached) in SyS. Translational data showed afami-cel mechanistically drives tumoral infiltration of activated and proliferative cytotoxic T cells, shifting balance from an immunosuppressive to pro-immune tumor microenvironment [81]. Given favorable sarcoma results, a phase II trial (SPEARHEAD-1) in HLA-A*02–eligible patients with advanced MAGE-A4–expressing SyS or MRCLS was opened. As of August 30, 2023, primary efficacy endpoint was met, indicating afami-cel is efficacious in these heavily pre-treated patients, with ORR per RECIST v1.1 by independent review and median DOR of 39 % (17 PRs) and 11.6 months (95 % CI 4.4–18.0), respectively, in 44 patients with SyS, and 25 % (two PRs) and 4.2 months (95 % CI 2.9–5.5) in eight patients with MRCLS. Safety findings aligned with those of the phase I trial except there were no treatment-related fatalities, with transient low-grade CRS and grade ≥ 3 cytopenias occurring in most patients [82]. ICANS occurred in ≤ 5 % of patients in the phase I and II afami-cel trials [81,82]. Afami-cel received accelerated approval in August 2024 from the FDA for treatment of adults with unresectable or metastatic SyS who have received prior chemotherapy; are HLA-A*02:01P, −A*02:02P, −A*02:03P or −A*02:06P positive; and whose tumor expresses MAGE-A4 as determined by FDA-approved diagnostics.

SURPASS is a phase I clinical trial of the next-generation product uza-cel in HLA-A*02–eligible patients with advanced MAGE-A4–expressing cancers across multiple solid tumor indications (NCT04044859). At latest data cutoff (August 14, 2023), 46 patients had received uza-cel as monotherapy and ten in combination with the programmed cell death protein 1 (PD-1) inhibitor nivolumab. AEs appeared consistent with those experienced by people with advanced cancers undergoing chemotherapy, immunotherapy and/or adoptive cell therapy. CRS occurred in 42 (75 %) patients, cytopenia at 4 weeks post infusion occurred in 15 (27 %) patients and nine (16 %) patients experienced ICANS. There were three possibly treatment-related fatalities: a 60-year-old patient with ovarian cancer who died of pneumonia and CRS, a 69-year-old patient with ovarian cancer who died of myositis > 8 months post T-cell infusion and a 71-year-old patient with esophageal adenocarcinoma and history of anemia who died of pancytopenia. ORR per RECIST v1.1 by investigator review was 35 % (95 % CI 21–50) in patients receiving monotherapy, with median DOR of 21 weeks (95 % CI 12–38). Clinical responses were associated with strong evidence of tumor infiltration by endogenous and engineered T cells, broad immune engagement and anti–MAGE-A4 tumor activity. A particularly encouraging ORR of 50 % (95 % CI 30–70) in the subset of 26 patients with ovarian, head and neck and urothelial cancers led to these being indications of focus [83]. Consequently, expansion SURPASS cohorts enrolling patients with head and neck or urothelial cancers administering uza-cel in combination with pembrolizumab as an early-line therapy [84,85], and a phase II trial (SURPASS-3) of uza-cel as monotherapy or in combination with nivolumab in patients with MAGE-A4–positive platinum-resistant ovarian cancer [86], were opened. Of note, a protocol amendment implemented during the phase I afami-cel study increased threshold of MAGE-A4 expression required for eligibility to require ≥ 30 % of tumor cells stain at 2 + or 3 + intensity by IHC. This threshold was also applied to SPEARHEAD-1 and SURPASS.

Several additional strategies to enhance efficacy and improve safety of TCR T-cell therapies targeting CTAs are under clinical investigation. Endogenous TCR-silencing mechanisms are purported to overcome potential decreased TCR surface expression and reduced biological activity [87], in addition to mediating self-reactivity derived from mixed TCR dimers formed from introduced and endogenous TCR α/β chains [88]. A first-in-human phase I clinical trial of endogenous TCR-silenced and affinity-enhanced NY-ESO-1 TCR T cells reported clinical responses with acceptable safety, except for grade 3 lung injury in a patient with SyS due to lung infiltration of TCR T cells [89]. To ensure maintenance of T-cell functioning against the backdrop of the immunosuppressive tumor microenvironment, combining TCR T-cell therapy with immune checkpoint inhibitors such as nivolumab or pembrolizumab, which inhibit the programmed death/ligand 1 (PD/L1) axis, is currently being evaluated in clinic (as described above, e.g., in SURPASS). Findings such as PD-1 inhibition enhancing *in vivo* efficacy of CAR T cells in a xenograft model of advanced thyroid cancer support rationale for combination of adoptive cell therapies with immune checkpoint inhibitors [90]. Interestingly, translational data from SPEARHEAD-1 and SURPASS suggest not only do TCR T cells infiltrate target tumors, they also facilitate endogenous T cell recruitment and wider immune response activation [83,91]. High MAGE-A4 expression was significantly associated with response to ipilimumab/nivolumab in a series of 13 mucosal melanomas [92]. A case report also suggested low-dose radiotherapy could enhance clinical benefits of TCR T-cell therapy [93]. In addition, engineered T cells targeting multiple antigens (PRAME, NY-ESO-1, MAGE-A4, SSX2) in a patient with refractory breast cancer were well tolerated and induced disease stabilization [94]. As trials continue to treat patients with TCR T-cell therapies and follow-up time increases, more will be learned about possible rare side effects and how to manage potential toxicities associated with treatment (e.g., a case of lymphoproliferative disorder following afami-cel infusion [95]).

## Current limitations to MAGE-A4–targeted TCR T-cell therapies

For current TCR T-cell therapies to function, patients must express the appropriate HLA type complexed with the MAGE-A4 epitope that the TCR was engineered to target. Not all cancers express MAGE-A4, and not all tumors express MAGE-A4 at levels high enough to be considered for TCR T-cell therapies, depending on thresholds used to define “positivity” for each therapy. Furthermore, impact of MAGE-A4 expression on prognosis is not clear. Alternatively, HLA-A*02 genotypes consisting of HLA-A*02:01, HLA-A*02:05 and HLA-A*02:06, compared with other HLA genotypes, were shown to be associated with shorter overall survival in SyS [96]; similar patterns, identified retrospectively, have also been noted in other cancers [97,98]. Thus, tumor type, MAGE-A4 expression and HLA type are carefully considered criteria in current clinical trials that restrict eligibility, thereby reducing the number of people who can potentially benefit from treatment.

Even for eligible patients, response rates and durations of MAGE-A4–targeted TCR T-cell therapies could be improved, as discussed above. Once infused, engineered therapeutic T cells have to engage with the same elements as a native T cell, including an immunologically cold tumor microenvironment with regulatory T cells, inhibitory macrophages and myeloid-derived suppressor cells. The phenotypic composition of infused T cells is also thought to have an impact, whereby infusion of predominantly late memory cells gives rise to more T-cell exhaustion and less persistence than infusion with stem cell–like or early memory phenotype T cells [99]. Loss or reduction of target antigen expression is also a potential resistance mechanism. However, to date, loss or reduction of antigen presentation machinery seems to be a more common mechanism of resistance to TCR T-cell therapies targeting CTAs. Loss of HLA-restricted antigen presentation may occur through epigenetic silencing or deletion/mutations of *HLA* genes, loss of heterozygosity and mutations in antigen presentation or β−2 microglobulin genes [99]. In patient samples taken at progression after treatment with letetresgene autoleucel, a TCR T-cell therapy targeting NY-ESO-1, expression of NY-ESO-1 remained high, but there was a decrease in *HLA-A* and antigen presentation gene expression [100].

The patient’s journey must be considered in any treatment modality. TCR T-cell therapy involves collection of a patient’s T cells via apheresis, manufacturing of MAGE-A4–targeted T cells and re-infusion back into the patient, usually following LD chemotherapy. The amount of time from apheresis to engineered T-cell infusion may limit benefits of treatment due to potential increases in tumor burden during that time. Future delivery mechanisms, including on-site high-speed manufacturing [101], allogeneic T-cell products or *in vivo* TCR delivery, offer potential to minimize lead time from identification to treatment and increase reach of these therapies. In addition, serious AEs have occurred despite preclinical testing for off-target effects, indicating typical cell culture systems may not always be sufficient to comprehensively assess risks for toxicity preclinically. Furthermore, potential for fatalities is not limited to off-target effects, and management of AEs associated with mechanisms of TCR T-cell therapies targeting MAGE-A4 must be anticipated.

## Other immunotherapies targeting MAGE-A4

Given the acknowledged appeal of MAGE-A4 as a target for immunotherapy for various solid tumors, combined with potential limitations described above, alternative targeting methods are also under active investigation that have varying levels of similarity to engineered TCR T-cell therapies. For example, IMC-C103C (MAGE-A4 × CD3) is an ‘ImmTAC’ (immune-mobilizing monoclonal TCR against cancer), a bispecific protein consisting of a modified TCR targeting MAGE-A4 fused to an anti-CD3 single-chain variable fragment effector domain, designed to induce T-cell activity regardless of T-cell specificity [102]. Preliminary data from a phase I dose-escalation trial of IMC-C103C (NCT03973333) in 42 HLA-A*02:01 patients with advanced solid tumors showed a manageable AE profile, including CRS and neutropenia, with no treatment-related fatalities. Clinical activity was observed in four patients with platinum-resistant ovarian cancer (two PRs, two reductions in target lesions), all of whom had low MAGE-A4 expression [103]. In an expansion cohort of the same trial enrolling patients with pre-treated ovarian cancer, a similar AE profile was observed, and six of 15 patients with MAGE-A4 expression had tumor shrinkage, with one PR [104]. This trial has now been terminated. IMA401 is a T-cell–engaging receptor comprising a TCR domain targeting an HLA-A*02:01–restricted MAGE-A4/A8 antigen, a T-cell recruiting antibody and an Fc domain. IMA401 showed a manageable safety profile and indications of anti-tumor activity in an ongoing phase I trial (NCT05359445); three of 20 patients with recurrent/refractory solid tumors had confirmed PRs [105]. Other examples include the CHP-MAGE-A4 cancer vaccine, comprising cholesteryl pullulan (CHP) and MAGE-A4 nanoparticles, which was administered to 15 patients with advanced esophageal, stomach or lung cancer, two of which had SD. Four of 15 patients exhibited a MAGE-A4–specific humoral immune response, and these patients had a longer overall survival than those without an immune response [106].

Although clinical utility has not been demonstrated, early preclinical mouse models of allogeneic TCR T-cell therapy targeting NY-ESO-1 with additional small interfering RNAs that downregulate endogenous TCR and HLA expression showed disease control without causing graft versus host disease, showing potential of an approach where individual patient cell manufacturing processes are not required [107]. Preclinical studies have also shown *in vitro* and *in vivo* anti-tumor activity of T cells engineered with a “TCR-like” CAR receptor targeting MAGE-A4 [108]. A phase I trial of MU-MA402C (jRCT2043210077), a second-generation “TCR-like” CAR T-cell therapy targeting a MAGE-A4 antigen in complex with HLA-A*02:01, in patients with MAGE-A4–positive advanced solid tumors is reported to be recruiting [109]. Preclinical data suggest ZI-MA4–1a, allogeneic natural killer cells transduced to express the CD3 complex, CD8 co-receptor and an affinity-enhanced TCR directed toward a MAGE-A4/HLA complex, could have anti-tumor activity [110].

## Conclusion

Various solid tumors overexpress CTAs; however, a robust anti-tumor immune response triggered by these antigens is generally suppressed. MAGE-A4 is an attractive target for immunotherapy due to its expression across cancer types, cancer specificity, possible oncogenic activity and immunogenicity. Engineered TCR T-cell therapies potentially show some key advantages over other immunotherapies such as TIL and CAR T-cell therapies, including the ability to target intracellular antigens. Increased potency of engineered MAGE-A4–targeted TCR T-cell therapies has led to promising preliminary clinical results and FDA approval of one therapy. Furthermore, overall safety findings have indicated a manageable risk profile, but extensive preclinical characterization of putative off-target, and potential on-target but off-tumor, effects should be applied, and clinical toxicity should be monitored closely to avoid fatal outcomes.

## Figures and Tables

**Table 1:** Summary of clinical findings of trials of TCR T-cell therapies targeting MAGE-A4.

Trial identifier	Phase	Tumor type	Number of patients	Treatment	Status and key findings

UMIN000002395 [80]	I	Recurrent/metastatic esophageal carcinoma	10	TCR-transduced T cells followed by MAGE-A4 peptide vaccinations	Completed. Minimal AEs, some grade 1 skin reactions. Disease progression per RECIST v1.0 in seven patients within 2 months; three patients survived > 27 months
NCT03132922 [81]	I	HLA-A*02-eligible patients with MAGE-A4-expressing tumors of nine types	38	Lymphodepletion chemotherapy followed by a single infusion of afami-cel	Completed. AEs included hematologic toxicities and CRS, with two possibly treatment-related fatalities. Overall response rate per RECIST v1.1: 24 % (9/38), with 7/16 (44 %) PRs in synovial sarcoma and 2/22 PRs in all other cancers
NCT03247309	I	HLA-eligible patients with recurrent/ refractory solid tumors expressing MAGE- A4 and/or A8	7	Lymphodepletion chemotherapy followed by infusion of IMA201 (TCR-engineered autologous T cells) then low-dose interleukin 2	Completed, no results posted
NCT04044768 (SPEARHEAD-1) [82]	II	HLA-A*02-eligible patients with MAGE-A4-expressing advanced synovial sarcoma or myxoid/round cell liposarcoma	52 in cohort 1	Lymphodepletion chemotherapy followed by a single infusion of afami-cel	Recruitment closed and follow-up ongoing for cohorts 1 and 2; cohort 3 recruiting. The primary efficacy endpoint was met, with overall response rate per independent RECIST v1.1 in cohort 1 of 37 % overall, 39 % in synovial sarcoma and 25 % in myxoid/round cell liposarcoma. AEs included CRS and cytopenias, with no treatment-related deaths
NCT04044859 (SURPASS) [83]	I	HLA-A*02-eligible patients with advanced MAGE-A4-positive melanomas, synovial sarcomas, myxoid/round cell liposarcomas, ovarian, esophagogastric junction, esophageal, gastric, urothelial, head and neck or non-small cell lung cancers	Target: 120	Lymphodepletion chemotherapy followed by uza-cel monotherapy or in combination with nivolumab or pembrolizumab	Recruitment now closed. As of August 2023, AEs included CRS and cytopenias, with three deaths possibly treatment related; overall response rate per investigator RECIST v1.1 was 35 % in the monotherapy cohort
NCT01694472	I	HLA-A*24:02- and MAGE-A4-positive solid tumors that have failed standard therapies	Target: 15	Two infusions of MAGE-A4 TCR gene-modified T cells followed by two peptide vaccinations	Not recruiting, no results posted
NCT02096614	I	HLA-A*24:02- and MAGE-A4-positive advanced solid tumors	18	Lymphodepletion chemotherapy followed by TBI-1201 (MAGE- A4-specific TCR gene-transferred T lymphocytes)	Completed (April 2014-March 2021), no results posted
NCT04752358 (SURPASS-2) [111]	II	HLA-A*02-eligible patients with MAGE-A4-positive advanced esophageal or esophagogastric junction cancers	Target: 45	Lymphodepletion chemotherapy followed by uza-cel monotherapy	Recruitment closed in 2022 after enrolling three patients
NCT06170294	I	Advanced solid tumors	Target: 20	Lymphodepletion chemotherapy followed by JWTCR001 (autologous humanized anti-MAGE-A4 TCR- engineered T cells)	Recruiting, start date January 1, 2024
NCT05601752 (SURPASS-3) [86]	II	HLA-A*02-eligible patients with MAGE-A4-positive platinum-resistant ovarian cancer	Target: 66	Lymphodepletion chemotherapy followed by uza-cel monotherapy or in combination with nivolumab	Recruiting closed, start date June 26, 2023
NCT05642455 (SPEARHEAD-3)	I/II	HLA-A*02-eligible patients aged 2–21 years with MAGE-A4-expressing advanced synovial sarcoma, MPNST, neuroblastoma or osteosarcoma	Target: 20	Lymphodepletion chemotherapy followed by a single infusion of afami-cel	Recruiting, start date September 1, 2023

AE: adverse event; afami-cel: afamitresgene autoleucel; CRS: cytokine release syndrome; HLA: human leukocyte antigen; MAGE-A4: melanoma-associated antigen A4; MPNST: malignant peripheral nerve sheath tumor; PR: partial response; RECIST; Response evaluation criteria in solid tumors; TCR: T-cell receptor; uza-cel: uza-tresgene autoleucel.

## Abbreviations:

AE: adverse event
afami-cel: afamitresgene autoleucel
CAR: chimeric antigen receptor
CCL19: C-C motif chemokine ligand 19
CI: confidence interval
CR: complete response
CRS: cytokine release syndrome
CTA: cancer/testis antigen
CTL: cytotoxic T lymphocyte
DOR: duration of response
HLA: human leukocyte antigen
ICANS: immune effector cell-associated neurotoxicity
IFNγ: interferon gamma
IHC: immunohistochemistry
IL-7: interleukin 7
LD: lymphodepletion
MAGE: melanoma-associated antigen
MHD: MAGE homology domain
MRCLS: myxoid/round cell liposarcoma
mRNA: messenger RNA
NY-ESO-1: New York esophageal squamous cell carcinoma 1
ORR: overall response rate
PD-1: programmed cell death protein 1
PD-L1: programmed death-ligand 1
PR: partial response
RECIST: Response Evaluation Criteria in Solid Tumors
SD: stable disease
SyS: synovial sarcoma
TAA: tumor-associated antigen
TCR: T-cell receptor
TIL: tumor-infiltrating lymphocyte
TLS: *trans*-lesion synthesis
uza-cel: uzatresgene autoleucel
